# Cannabinoids: A Cause of Severe Bradycardia

**DOI:** 10.7759/cureus.16560

**Published:** 2021-07-22

**Authors:** Filipa Guimarães, João Camões, Marta Pereira, Rui Araujo

**Affiliations:** 1 Internal Medicine, Matosinhos Local Health Unit, Pedro Hispano Hospital, Porto, PRT; 2 Intensive Care Unit, Matosinhos Local Health Unit, Pedro Hispano Hospital, Porto, PRT

**Keywords:** bradycardia, cannabinoids, drug induced bradycardia, adenosine receptors, cbd products

## Abstract

The use of recreational cannabis affects almost all countries in the world, being illegal in most of them. Cannabis formulations include several compounds, with cannabidiol (CBD) being one of the best studied. One of its effects is its action in adenosine (anti-arrhythmic) receptors.

We present the case of a 39-year-old male with chronic anxiety and regular inhaled cannabis consumption who reported to the emergency department (ED) after a first use of inhaled CBD. While in the ED, the patient presented with four episodes of syncope. EKG was performed and an extreme bradycardia with a sinus pause of 13.8 seconds was observed.

Marijuana consumption is associated with various clinical manifestations, such as bradycardia, sinus pauses, and orthostatic hypotension. The tropism of this toxic for adenosine receptors can induce severe bradycardia, which can be life-threatening.

## Introduction

The use of recreational cannabis affects almost all countries in the world, being illegal in most of them. In Portugal, limited cannabis possession is decriminalized since 2001 [1].

Cannabis formulations include several compounds, with different impacts in their physical and psychological effects. The best studied are cannabidiol (CBD) and tetrahydrocannabinol (THC). The former is characterized by the absence of CBD action on cannabinoid receptors and, therefore, free of psychoactive effects (mainly euphoria) that characterize THC effects. As so, CBD is routinely used in medical prescription [2].

The effects of CBD have been progressively described over the last years, highlighting its action in adenosine (anti-arrhythmic), tumor necrosis factor alpha (anti-inflammatory), and opioid (analgesic) receptors [3]. There are scarce reports of side effects of CBD and they emerge mainly from animal studies and case reports, but sinus bradycardia and asystole have been reported [4].

## Case presentation

**Figure 1:**
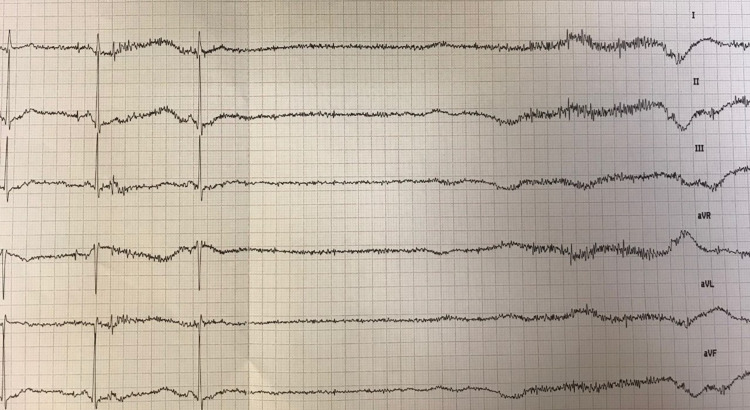
Severe bradycardia Sinus pause of 13.8 seconds

We present the case of a 39-year-old male with chronic anxiety and regular consumption of inhaled cannabis who reported to the emergency department after 12 hours of a first use of inhaled CBD. The patient reported an urticarial rash on the lower limbs and abdomen, without other pertinent symptoms or changes in initial clinical examination. No relevant epidemiological context or simultaneous medicines were reported. Intravenous steroids and clemastin were administered, with complete resolution of the rash. While in the emergency department, the patient presented with four episodes of syncope with prodromes (pallor and diaphoresis). These symptoms occurred with orthostatic position and were not accompanied by hypotension. EKG was performed in supine, sitting, and orthostatic position and, respectively, revealed a sinus rhythm of 70 bpm, 50 bpm, and extreme bradycardia with a sinus pause of 13.8 seconds (Figure 1), with clinical recurrence of syncope.

Initial laboratory results (including thyroid function) showed no major changes, and an echocardiogram revealed a normal biventricular function, without valvular abnormalities. The patient was monitored in the emergency department for the following 12 hours. He progressively tolerated elevation of bedhead and 24 hours later was standing, without clinical recurrence of symptoms or dysrhythmic events. The patient was discharged and interrupted CBD consumption but maintained cannabis use. After six months of follow-up, no clinical recurrence of symptoms was reported.

## Discussion

Sinus node disease is characterized by the inability of the heart to perform its pacemaker function, resulting in periods of inappropriate heart rhythm, which can be intermittent or persistent. The causes of isolated sinus dysfunction are vast and include intrinsic (infiltrative, degenerative, or inflammatory) diseases but also extrinsic contributors, such as metabolic or drug-induced [5].

The association of cannabis use and increased risk of cardiac dysrhythmia is supported by plausible physiological mechanisms. The primary psychoactive cannabinoid component is THC with its metabolites 11-hydroxy-tetrahydrocannabinol and 11-nor-9-carboxy-tetrahydrocannabinol. CBD and cannabinol are other active cannabinoids. Smoking is the most frequent form of exposure leading to rapid uptake and transfer from lungs to brain. Although coronary blood flow represents only 10% of systemic blood flow, these compounds can be retained within the heart for days, which may justify the cardiac tropism affecting the sinus node [6].

Marijuana consumption is associated with various clinical manifestations, such as bradycardia, sinus pauses, and orthostatic hypotension [7]. Although rare, vasovagal syncope has been described as one of the side effects of cannabis and this case demonstrates an example of that, leading to a sinus pause. The authors believe that there are two possible explanations to this sinus pause; on one hand because of extreme vagal tone [8], and on the other, for its tropism for adenosine receptors. 

Although decriminalized, recreational marijuana consumption is illegal in Portugal, but some legal compounds have been recently launched in the market, most of them containing CBD. In the case presented, the patient used a CBD-based compound for the first time and had syncope with a recorded sinus pause larger than 13 seconds. CBD is a substance with tropism for adenosine receptors (which THC has not), which may justify the rhythm disturbance described. Since CBD has a washout time of 18-32 hours [9], we monitored the patient for 36 hours to assure there were no recurrent clinical or electrical manifestations. Other metabolic and structural causes were excluded and, after six months of CBD cessation, no symptoms have been reported.

## Conclusions

CBD consumption is anticipated to increase for medicinal use in many countries. Cannabis use is associated with increased risk of cardiac dysrhythmia, which is rare but may be life-threatening. Clinicians should inquire about acute and chronic cannabis use in their patients presenting with tachycardia, bradycardia, dysrhythmia, chest pain, and/or unexplained syncope. The tropism of this toxic for adenosine receptors can induce severe bradycardia, which can be life-threatening, a fact that needs to be widely shared with health system and the public.
